# Automatic Detection of Human Maxillofacial Tumors by Using Thermal Imaging: A Preliminary Study

**DOI:** 10.3390/s22051985

**Published:** 2022-03-03

**Authors:** Diana Mačianskytė, Rimas Adaškevičius

**Affiliations:** 1 The Clinic of Radiology, Lithuanian University of Health Sciences, LT-50009 Kaunas, Lithuania; 2 Department of Electrical Power Systems, Kaunas University of Technology, LT-51368 Kaunas, Lithuania; rimas.adaskevicius@ktu.lt

**Keywords:** infrared thermal image, CT, machine learning algorithm, *k*NN classifier, orofacial/maxillofacial tumor

## Abstract

Traditional computed tomography (CT) delivers a relatively high dose of radiation to the patient and cannot be used as a method for screening of pathologies. Instead, infrared thermography (IRT) might help in the detection of pathologies, but interpreting thermal imaging (TI) is difficult even for the expert. The main objective of this work is to present a new, automated IRT method capable to discern the absence or presence of tumor in the orofacial/maxillofacial region of patients. We evaluated the use of a special feature vector extracted from face and mouth cavity thermograms in classifying TIs against the absence/presence of tumor (*n* = 23 patients per group). Eight statistical features extracted from TI were used in a *k*-nearest neighbor (*k*NN) classifier. Classification accuracy of *k*NN was evaluated by CT, and by creating a vector with the true class labels for TIs. The presented algorithm, constructed from a training data set, gives good results of classification accuracy of *k*NN: sensitivity of 77.9%, specificity of 94.9%, and accuracy of 94.1%. The new algorithm exhibited almost the same accuracy in detecting the absence/presence of tumor as CT, and is a proof-of-principle that IRT could be useful as an additional reliable screening tool for detecting orofacial/maxillofacial tumors.

## 1. Introduction

Infrared radiation from human skin can be described by an exponential function of the surface temperature, which is influenced by the level of blood perfusion in the skin [1]. Unlike images created by X-rays or proton activation through magnetic resonance, thermal imaging (TI) is not based on morphological analysis. The technique provides only a map of the distribution of temperatures on the surface of the object imaged.

Infrared thermography (IRT), due to its apparent simplicity, is a feasible diagnostic tool in medicine and has been used in various fields for analyzing physiological and/or pathophysiological functions causing skin temperature changes for over 30 years [2] (for review see [3,4,5,6,7]). It is a non-invasive, contactless, passive, and noxious radiation-free technique that does not cause patient discomfort. However, it is still not widely used in routine clinical diagnosis, largely because of the lack of clinically approved medical devices, standardized protocols for IRT in medicine and, importantly, because the desired information cannot be extracted easily.

Nowadays, the development of new generation IRT techniques using high-resolution cameras has allowed the wide use of TI for better diagnostic capabilities. TI obtained with modern technology may contain several thousands of temperature points and enables accurate thermal mapping of very subtle changes in skin surface temperature that are clinically significant.

Local skin temperature can be influenced by many diseases and/or pathologies (e.g., tumors, inflammation, infection, fever, chronic pain diseases, changes in nervous system function, etc.) as well as by psychophysiological events (for review see [5,6,7]). The skin surface temperature is always the sum of all thermal processes taking place under the skin, and an awareness of the underlying physiology must be considered when making clinical decisions. So, the tissue temperature of the area surrounding a pathological zone can be different from that of normal tissue, but various conditions can have opposite effects on the temperature (e.g., low in necrosis or ischemia, high in inflammation). For a healthy person, the thermogram shows uniform and symmetric variations in skin temperature, and differences in selected areas from side to side are very small (~0.2 °C). In contrast, pathological regions show abrupt variations in temperature [8,9]. Thermal symmetry in the face is a normal finding in healthy subjects. Asymmetrical distributions of temperature as well as the presence of hot or cold spots are known to be strong indicators of an underlying dysfunction [9].

At present, IRT is most often used for the diagnosis and treatment monitoring of breast cancer, skin melanoma, and other pathologies [1,10,11]. However, it is also of potential importance for evaluating the development of pathologies in the early stages. Early detection can facilitate the subsequent clinical management of patients. In order to be practically relevant, systems of automated and computer-assisted IRT interpretation have to be developed and trained. Therefore, the application of machine learning (ML) tools to detect key features from complex datasets might be helpful [12] (for review see [13]).

The use of automated image analysis techniques is of particular importance in the orofacial and maxillofacial regions [2]; because their elaborate anatomical structure, it is very difficult to detect pathology (e.g., tumor) in the early stages. Usually, the patients are directed to other diagnostic methods, often in late stages, when the orofacial/maxillofacial tumor is already advanced in size and causes specific symptoms. To detect the tumor timely, potential patients should be examined before they have any complaints. This would imply that all healthy subjects undergoing preventive medical examination should be directed to CT. However, due to the high risk of radiation and its high costs, CT cannot be used as a screening method. Instead, ML IRT can be used as a non-invasive and radiation-free routine diagnostic method, which by detecting early temperature changes in the affected area, could enable to find the presence of possible tumors, and to timely direct the patient to further investigation. However, there are currently no screening ML IRT tools for automatic classification of human face and mouth cavity thermograms.

Considering the information presented above, the aim of this study was to develop a new automated ML TI method capable to discern the presence or absence of physiological abnormalities, due to tumors, by using skin surfaces temperature distribution in the orofacial/maxillofacial region. Our recent study showed that accurate detection of the facial TI, using an image processing algorithm based on the optimized region of interest (ROI) and image segmentation when searching for zones with asymmetrical temperature distribution, is a critical step for revealing maxillofacial tumors precisely [14]. In this paper, we present a new efficient algorithm, which is supplemented with the *k*NN classification scheme constructed from a training data set for TI features extraction and allows automatic recognition of tumors. The new algorithm exhibited almost the same accuracy in detecting tumor regions as CT and can be a fast method for efficiently classifying facial and mouth cavity TI of patients into non-tumor vs. tumor cases. Therefore, we provide proof of principle that IRT might be a complementary tool for doctors/radiologists to help in the screening of tumors, especially at the earlier stages.

## 2. Materials and Methods

### 2.1. Patients

Forty-six patients, who underwent a CT procedure in the orofacial/maxillofacial region during the years 2014 to 2016 at the Clinic of Radiology of the Hospital of the Lithuanian University of Health Sciences (LUHS), were enrolled in this study. Written informed consent was obtained before procedures. The inclusion criteria were: patients who had no orofacial/maxillofacial history, but who after clinical evaluation by different physicians (e.g., maxillofacial-oral surgery, otolaryngologists, etc.) were directed to a CT procedure for suspected pathology, and those who were evaluated before planning of tooth implantation. The overall characteristics of the patients are summarized in Table 1, which groups them depending on whether CT scan showed they had orofacial/maxillofacial tumor (T-group) or not (i.e., control patients, relative to the presence of tumor; NT-group).

The study was performed with the European Community guiding principles and with the approval of the Ethics Committee of Biomedical Research of Kaunas Region, Lithuania (no. BE-2-31, 3 June 2014).

### 2.2. Pre-Processing and Thermal Image Acquisition

The methods used for the TI data acquisition in patients have been described before [14]. In short, at the first step, a standardized TI was recorded for every patient prior to the CT scanning procedure, in order to avoid the subsequent influence of X-rays and/or contrast agents on the temperature profiles. All patients underwent the same thermographic protocol adjusted for clinical application, trying to minimize factors that might affect the TI data (medications, facial cosmetics, intake of tea/coffee, smoking, physical exercise, etc.). Before undertaking the measurements, all patients were thermo-equilibrated in the laboratory for 15 min [10,14]. During IRT measurements, all of them were instructed to sit as straight as possible. TI was taken from each patient’s face and mouth cavity (in frontal closed and open mouth projections). Surface temperature profiles of each patient were recorded and later analyzed.

The TI was captured using a commercially available FLIR E8 infrared thermal camera (FLIR Systems, Inc., Wilsonville, OR, USA), which was positioned one meter away from the patient’s face. TI was acquired using a resolution of 320 × 240 pixels, thermal sensitivity of ±0.06 °C, and a temperature range from −20 °C to +250 °C. The thermography measurements were performed in controlled environments: relative humidity of 60 ± 5%, and temperature of 22 ± 1 °C. Emissivity was set for the skin thickness value of 0.98 [14].

The TI captured by the IRT camera was subsequently transferred to a computer and analyzed using image processing software in Matlab R2014b (The MathWorks, Inc., Natick, MA, USA). The images were transformed into Matlab readable data files by ThermaCam Researcher 2.1 (FLIR Systems, Inc., Wilsonville, OR, USA).

### 2.3. CT Scanning Procedure

After TI acquisition, every patient was CT scanned (Toshiba Aquilion ONE, Canon Medical Systems, Otawara, Japan) using the same scanning parameters (thickness step of 1 mm), and CT-scan images were analyzed by a radiologist. CT was used as a gold standard exam with regard to identifying lesions, and according to CT-scan findings, the patients were divided into non-tumor (NT) and tumor (T) groups (Table 1).

### 2.4. Experimental Protocol for TI Exploration

**Figure 1:**
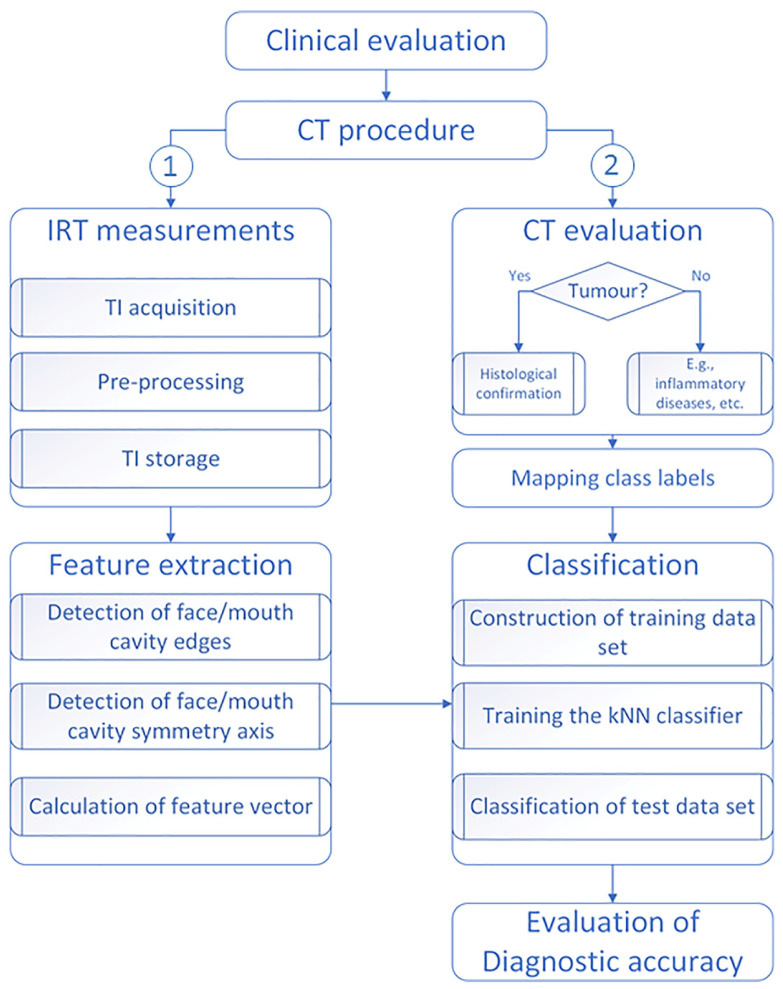
Flowchart showing the major steps of the study. Note: based on CT-scan findings, the TIs were classified as NT (0) and T (1) cases, i.e., the absence/presence of tumor lesions, respectively.

The proposed method comprises several steps, as presented in Figure 1.

As we have documented before [14], the algorithm includes: finding of orientation and position of the symmetry axis of the human face or of the mouth cavity, which helped calculate thermal features (see Table 2).

### 2.5. Detection of Human Face Edges

After acquisition and pre-processing of TI, the next step was the identification of human face image boundaries. For edge detection, the gradient-based approach was used. This approach is based on searching image points with a high gradient which corresponds to the points where the gradient magnitude is maximal. The edge is detected by looking for the maximum in the first derivative of the image.

**Figure 2:**
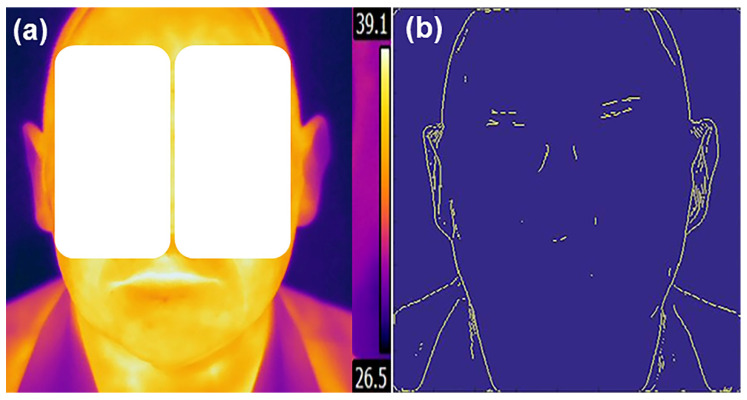
Original thermal image (**a**) and obtained human face edges (**b**).

TI gradient components in any direction were calculated using a convolution mask and the equations given in Appendix A.1, and pixels with the large gradient magnitude values were considered as edge pixels. The Prewitt method [15] was selected for its simplicity and its low computational load. An original human face’s TI and the detected edges are presented in Figure 2.

### 2.6. Detection of Human Face or Mouth Cavity Symmetry Axis

**Figure 3:**
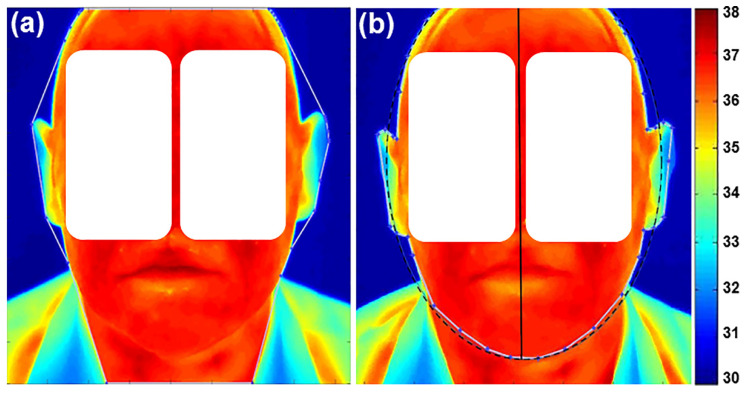
Estimated convex hull contour points (**a**) and symmetry axis (**b**) of a human face. The symmetry axis (continuous black line) is the major axis of the ellipse (dot line). Note: Normal color and thermal properties without abnormalities are presented. The corresponding scale bar is given on the right side in °C.

In order to find the orientation and position of a human face’s symmetry axis, only external face edge points are necessary. For the presentation of a human face’s external contour, the smallest convex polygons that contain all the edge points were determined using the convex hull algorithm [16] (see Appendix A and Appendix A.2). The convex hull of the edge image is a set of pixels included in the smallest convex polygon that surrounds all edge pixels and was used to describe the external boundary of the field, where temperature changes were analyzed. The selected points formed the convex hull (Figure 3a).

The ellipse best fitting the set of convex hull vertices, using least-squares criterion, was used for the identification of axis symmetry of the face (Figure 3b).

**Figure 4:**
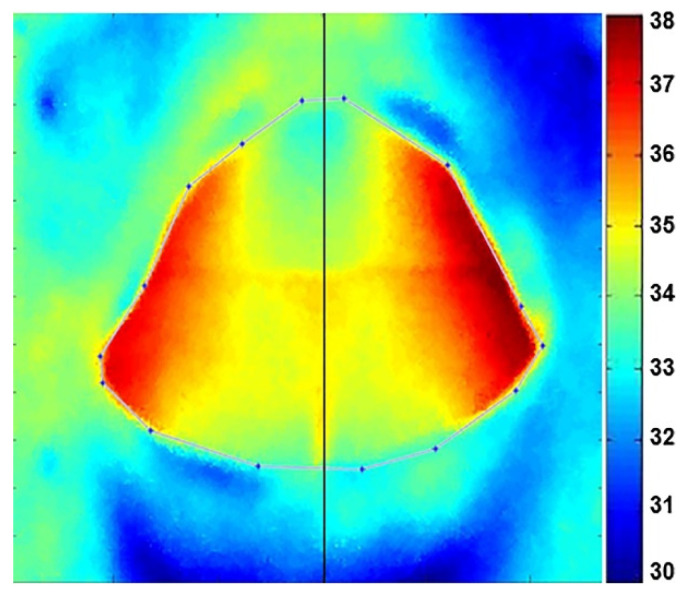
Estimated convex hull of mouth cavity contour points. Note: normal color and thermal properties without abnormalities are presented.

In the same way, the edges of the mouth cavity were extracted from the calculated convex hull. This symmetry axis was used for splitting the field of analysis into two independent mouth cavity regions of the TI (Figure 4) (see Appendix A and Appendix A.3).

### 2.7. Recognition of Tumor Region

For detecting tumors, using extracted areas of TI, the feature vector for each patient was created. The temperatures of individual pixels of four areas were used to calculate the features (∆*T_f_*, ∆*T_m_*, ∆*T_fmax_*, ∆*T_mmax_*, *n_f_*, *n_m_*, ∆*DEV_f_*, and ∆*DEV_m_*) (Table 2) for each patient (see Appendix A and Appendix A.4).

Of note: since in the study the majority of patients in the control group also had pathologies (e.g., inflammation) but no tumor; therefore, as was suggested before [9], the major inclusion criterion in the control group was to have thermal asymmetry (∆*T*) between both facial/mouth cavity sides not higher than 0.4 °C. Accordingly, when comparing the difference between temperatures of the left and right side of the NT class versus the T class, a temperature asymmetry higher than 0.4 °C was taken as being associated with the presence of abnormality [9]. Therefore, such a temperature difference can be used as a basis to form feature vector for a single patient.

The number of TI pixels of one given side of the face or mouth cavity having ΔT degree higher temperature than the maximal temperature of opposite side was calculated using histograms of both sides.

### 2.8. Training Procedure and Automatic Classification

For training of the system, each feature value has been calculated as average from five estimates of the same TI. This is done because the calculation of feature vector values requires estimating the contour of the face or the mouth cavity, which sometimes involved changing the positions of a few points. Feature vectors of NT-group patients (i.e., without tumors) and of T-group patients (i.e., with different orofacial/maxillofacial tumors) were stored as feature matrices in computer memory for the classification into classes, i.e., absence (0) or presence (1) of tumor, respectively.

In this study, the *k*-nearest neighbor (*k*NN) classification scheme [17] was used for the TIs, because the *k*NN has a tendency to work best on smaller data sets that do not have many features. The model of the *k*NN classifier is based on feature vectors and class labels from the training data set.

The *k*NN classifier is commonly based on the Euclidean distance between a test sample and the specified training samples:

(1) $$d=\sum_{n=1}^{p}\left(x_{n}^{test}-x_{n}^{train}\right)\;,$$

where p is the size of the feature vector, $x_{n}^{test}$ is the value of the sample vector representing features of test TI, $x_{n}^{train}$ is the values of the vector representing features extracted from TI, used for training.

During the classification stage, the vector values representing features extracted from TI of one patient are compared with the values in the feature matrix created during the training. Because the number of nearest neighbors has been selected as *k =* 1, then the patient data are simply assigned to the class of single nearest neighbor, representing the corresponding group of patients (NT or T).

In the study, such feature vectors were calculated for all patients. Thereafter, the dataset were randomly divided into two subsets for training and testing. The training subset involves only half of the data: 12 NT-images of patients without tumor lesions and 12 T-images of patients with tumor changes. The trained-test split procedure was repeated 12 times and different datasets were used for training and testing of the presented method.

The *k*NN algorithm assigns a category to observations in the test dataset by comparing them with the observations in the training dataset. Because we know the actual category of observations in the test dataset, the performance of the *k*NN model can be evaluated.

For estimating the correctness of classification/misclassification of the method presented here a confusion matrix was formed and the accuracy, sensitivity, and specificity values were calculated. Classification accuracy was evaluated by CT, and by creating a vector with the true class labels for obtained TI. Furthermore, the comparison of this vector with the results of classification obtained using was performed. The rate of correct solutions was selected as recognition accuracy parameter. It can be calculated as a ratio between the correctly classified TI to all the obtained data. The sensitivity of *k*NN classifier was calculated as the ratio between the number of correctly classified T-cases and the number of true T-cases. The specificity of the classifier was calculated as the ratio between the number of correctly classified NT-cases and the number of true NT-cases.

### 2.9. Statistical Analysis

All diagnostic data of the patients obtained by CT were classified either into NT-group (without tumors) or T-group (with tumors). The *t*-test was used to evaluate whether the values of a particular feature for NT-group are significantly different from values of the same feature for the T-group. If this holds, then the feature can be used to differentiate all data. This analysis is used for the analysis of a two-group experimental design. We used a significance level of *p* < 0.05 for statistical tests. The average temperatures of individual pixels of four human face areas and the independent samples *t*-tests of features were computed for each study group. All features were expressed as mean ± standard deviation (SD). The two-sample *t*-test analysis was performed in Matlab. In addition, a receiver operating characteristic analysis (ROC) was used for the comparison of diagnostic efficiency.

## 3. Results

### 3.1. Experimental Verification of the Method

**Figure 5:**
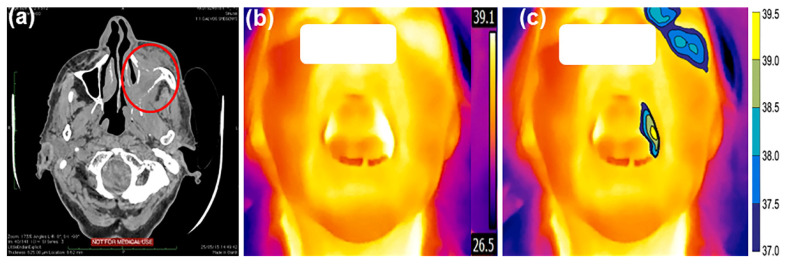
Mapping of a CT-scan image with TI without preprocessing and after segmentation. A CT-scan image (**a**) is presented in the axial plane and shows large infiltrative pathologic masses on the left side (marked by an oval ellipse). The TI of the same patient is presented in an open mouth position, and the area with higher temperature could be seen on the left side of the mouth cavity before (**b**) and after the segmentation (**c**) of images. The corresponding temperature scale bar is given on the right side in °C.

For each of the 46 patients, two frontal images of the face and of the mouth cavity were investigated. The mean temperature within each ROI was calculated and the difference between the affected region vs. the unaffected region in the opposite facial/mouth side was evaluated. As expected, temperature distribution between left and right parts of the human face as well as of the mouth cavity was symmetrical for TI of the NT-group (∆*T* ≤ 0.4 °C) (see Figure 3b and Figure 4, respectively) and matched well with the CT-scan findings (not illustrated), where no visible tumors in the orofacial/maxillofacial region were obtained. In contrast, in the T-group the size and location of tumor as revealed by CT coincided well with the higher temperature zones (∆*T* > 0.4 °C) obtained in the corresponding TI. Figure 5 shows a comparison of the images between the tumor area, detected with a CT (Figure 5a), and the presence of a changed thermal area, obtained with IRT (Figure 5b,c), for the same patient. Note that when the tumor is large in size, the affected zone could be identified easily from the TI without any additional processing [14].

Segmentation, as presented in Figure 5c, is used to represent the area of the tumor in a more meaningful and easy-to-analyze way. The filled contours are used to assign a color to each pixel in the image so that pixels of the same color have a temperature within a specified narrow range.

### 3.2. Evaluation of Diagnostic Accuracy

Table 3 provides the mean values of these features for the two classification classes, NT and T, representing data in the absence/presence of tumors. 

After classification of all TI into two classes, the following performance values with the presented method were obtained using equations described previously [18]: sensitivity of 77.9%, specificity of 94.9%, and accuracy of 94.1%.

**Figure 6:**
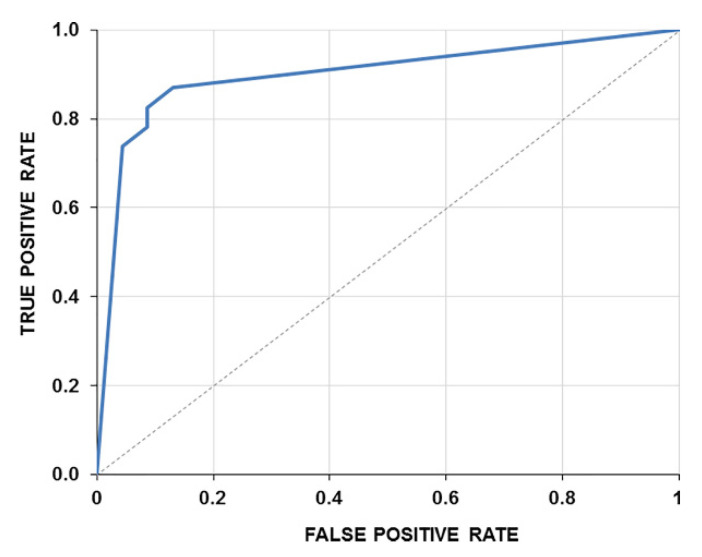
The ROC curve of the statistical test built. The true positive rate is plotted as a function of the false-positive rate. The area under the ROC curve (AUC) combines measures of sensitivity and specificity. The larger the AUC, the better the performance of the diagnostic test to correctly pick up cases with and without pathologies. AUC is 0.8769.

In addition, for the verification of the formed confusion matrix, the associated receiver operating characteristic curve (ROC), as presented in Figure 6, was built. The area under the ROC curve indicates the overall performance of a diagnostic test in terms of its accuracy at various diagnostic thresholds used to discriminate cases and non-cases of a disease [19].

## 4. Discussion

This paper aimed to provide a proof-of-principle that a new automated ML TI algorithm is capable to help discern the presence and absence of tumors using skin surfaces temperature distribution in the human face. Since in medicine, careful interpretation of results is essential, we performed the comparison between CT findings and TI obtained with IRT, to identify tumors in the selected orofacial/maxillofacial region. Our study rests on the selected threshold for ∆*T* values when describing the level of pathology, i.e., lower or higher than 0.4 °C, corresponding to the absence or presence of the pathology, respectively. Calculations performed in our study confirmed such threshold conditions as was suggested before [9]. In our study, if we used a threshold lower than 0.4 °C (e.g., 0.35 °C), some of the “control“ patients were ascribed to pathology (more false-positive cases). In contrast, when the threshold was higher than 0.4 °C, then some of tumor patients were ascribed to “control” patients (more false-positive cases). Accordingly, this confirmed that the optimal threshold under our experimental conditions is similar to the one used previously by others [9].

The proposed algorithm revealed that the presence of tumors defined by CT perfectly corresponded with the higher temperature zones defined by the IRT. Here, the employed automated image analysis technique provided highly reproducible findings, and no patients with orofacial/maxillofacial tumors were misdiagnosed. This suggests that ML IRT imaging examination is efficacious and, possibly, could be used as a non-invasive routine diagnostic method to detect early temperature changes in the affected region of the human face.

IRT as well as the asymmetry technique, by measuring regional temperature, which is a natural index of physiological function, can provide crucial information on the presence, location, and severity of alterations due to tumor. Therefore, on the one hand, due to its safety and exact recognition of tumor zones, the IRT method could be a useful supplement to the conventional method and may help in the medical as well as in the healthcare fields for the initial examination of patients. On the other hand, IRT still is not applied in the routine diagnosis of patients in the clinic, due to lack of certified thermal tools available to physicians, absence of standards for setting an appropriate ROI, of unified protocols, and overall requirements for the qualification of medical thermologists for clear interpretation of obtained TI. In addition, there are a lot of other reasons why thermal imaging is not applied in clinical routines, especially due to artefacts caused by facial hair, skin conditions, scars, “blushing” effect, asymmetrical distribution of adipose tissues, facial paralysis, veins near the superficial layer of the skin, etc. To date, IRT has been adopted extensively in the early detection of breast cancer [20,21,22,23], but for orofacial tumor detection, the available evidence is limited and weak [24,25]. It should also be underlined that according to the newest healthcare guidelines [26,27], current evidence does not support the routine use of thermography as a screening procedure in either field of clinical diagnosis. This is because there is insufficient evidence to conclude that IRT (including magnetic resonance thermography and temperature gradient studies) benefits tumor evaluation and because all technical findings require to complete the final interpretation, which would prevent overdiagnosis and misinterpretation of the TI findings.

In this respect, ML IRT can serve as a versatile screening method for giving the correct answers in the diagnosis of absence or presence of tumors, avoiding very subjective interpretation of the images. Major ML technical advances have been accomplished in recent years with the development of appropriate algorithms for the automatic detection of abnormalities [28,29,30] (for review see [12]), which help in interpreting imaging findings as well as minimizing errors or misinterpretation of images. Nevertheless, to our knowledge, nowadays there are no appropriate ML IRT algorithms for the screening of tumors in the facial region, especially for the detection of tumors in early stages. Our automated analysis algorithm could possibly be used for this early detection of both orofacial and maxillofacial tumors, but more extensive studies will be needed to test this. The algorithm has been successfully designed to recognize both trained and untrained data. The presented algorithm for extracting features of TI gives good results of the *k*-nearest neighbor classification scheme. The classification accuracy is 94.1%. The presented method is simple, exhibits quite good performance, and does not require significant computational resources, allowing data collection for long time periods using a small battery as a power source.

In summary, the predictive power of ML IRT has not been fully exploited. Beyond obtaining a good classification accuracy, for more accurate recognition the artificial neural network or support vector machine (SVM) classification algorithms can be used. A primary limitation of the study might be related to the small input data amount. The larger the training complex, the more accurate the existence of the tumor can be predicted. Another limitation can be linked with the absence of a real control group, including human subjects without inflammation, infection or immune reactions, as would be expected for healthy volunteers. One more limitation can be linked to a late stage of the pathology because in most of the patients the abnormal structures were already advanced in size and caused specific symptoms. To detect the pathology in the early stages, patients should be examined before they have any complaints. Unfortunately, at the moment only a few patients passed this criterion. Therefore, data from larger groups of patients are needed before the algorithm could be used as a non-invasive routine diagnostic method, especially for the early detection of tumors. As future work, more cases will be added to the classification states, and more experimental tests with more persons, including healthy volunteers, will be carried out.

## 5. Conclusions

We present a novel approach to the problem of recognition of orofacial/maxillofacial tumors using TI. The approach reveals that the new algorithm exhibited nearly the same accuracy in detecting tumor-affected regions as CT. Therefore, this is a proof-of-principle that the presented algorithm could be a fast method for efficiently classifying facial and mouth cavity images into non-tumor and/or tumor cases. The proposed algorithm is promising as a useful and reliable screening tool and, possibly, for the early detection of tumors in the orofacial/maxillofacial region. In addition, it may assist in providing a clearer interpretation of TI, avoiding reliance on subjective criteria.

## Figures and Tables

**Table 1:** Clinical characteristics of the patients.

Patient Data	NT-Group	T-Group
Age range (years)	18–78	18–86
Mean age (years) ± SEM	49.5 ± 3.5	57.1 ± 2.8
Female, *n* (%)	17 (73.9)	8 (34.8)
Male, *n* (%)	6 (26.1)	15 (65.2)
Total, *n* (%)	23 (100)	23 (100)
Anatomical regions		
Detected by CT		
Right-sided, *n* (%)	0 (0)	10 (43.5)
Left-sided, *n* (%)	0 (0)	12 (52.2)
Both-sided, *n* (%)	0 (0)	1 (4.3)

NT—non-tumor group, which includes patients with inflammatory diseases and with lymphatic nodes diseases of the maxillofacial area, and having no tumor lesions on CT evaluation; T—tumor group, which includes patients with different orofacial/maxillofacial tumors detected by CT and confirmed histopathologically.

**Table 2:** Terminology of statistical features extracted from TI.

Features	Definition
$\Delta T_{f}\;$ (°C)	The difference between mean temperatures of R vs. L sides of the face.
$\Delta T_{m}$ (°C)	The difference between mean temperatures of R vs. L sides of the mouth cavity.
$\Delta T_{fmax}$ (°C)	The difference between max temperatures of R vs. L sides of the face.
$\Delta T_{mmax}$ (°C)	The difference between max temperatures of R vs. L sides of the mouth cavity.
$n_{f}$	The number of pixels in TI of the R (L) side of the face having ΔT degree higher temperature than the max temperature of opposite face side.
$n_{m}$	The number of pixels in TI of the R (L) side of the mouth cavity having ΔT degree higher temperature than the max temperature of opposite mouth cavity side.
$\Delta DEV_{f}$	The difference of the absolute deviations of the temperature values of all pixels belonging to the R and L face side, respectively.
$\Delta DEV_{m}$	The difference of the absolute deviations of the temperature values of all pixels belonging to the R and L mouth cavity side, respectively.

R and L denote right and left side, respectively.

**Table 3:** Feature vectors formed for the TI of both NT (*n* = 23) and T (*n* = 23) groups.

Group	$\Delta t_{f}{,}^{\;}{}^{\circ}C$	$\Delta t_{fmax},\;{}^{\circ}C$	$n_{f}$	$\Delta DEV_{f}$	$\Delta t_{m}{,}^{\;}{}^{\circ}C$	$\Delta t_{mmax},\;{}^{\circ}C$	$n_{m}$	$\Delta DEV_{m}$
NT	0.14 ± 0.037	0.15 ± 0.038	0.086 ± 0.07	0.117 ± 0.128	0.16 ± 0.032	0.22 ± 0.036	0.304 ± 0.19	0.063 ± 0.015
T	0.23 ± 0.087	0.22 ± 0.062	2.48 ± 1.85	0.041 ± 0.012	0.46 ± 0.143	0.56 ± 0.169	48.57 ± 22.6	0.139 ± 0.043

NT- and T-patients without orofacial tumors and patients with obvious orofacial tumors, respectively; for definition of features (∆*T_f_*, ∆*T_m_*, ∆*T_fmax_*, ∆*T_mmax_*, *n_f_*, *n_m_*, ∆*DEV_f_*, and ∆*DEV_m_*) see Table 2. The significance of the difference between NT vs. T data was evaluated using confidence intervals. Note: only the mouth cavity data have statistical significance at a confidence level of 95%. However, at a confidence level of 90%, all data have statistical significance (not illustrated).

## Data Availability

Not applicable.

## Appendix A

### Appendix A.1. Calculation of TI Gradient Components

Each gradient component of the TI can be calculated using a convolution, and gives the value of the gradient in any direction as shown in the following relationships (Equations (A1) and (A2)), respectively [15]:

(A1) $$\nabla_{x}=I\left(x,y\right)*G_{x},$$

(A2) $$\nabla_{y}=I\left(x,y\right)*G_{y},$$

where *I(x*,*y)* is the temperature value of *(x*,*y)* pixel of the TI; * denotes the two-dimensional convolution operation, *G_x_* is the convolution mask along *x* axis; and *G_y_* is the convolution mask along y-axis, which can be described using Equations (A3) and (A4), respectively:

(A3) $$G_{x}=\left[\begin{array}{ccc} -1 & -1 & -1\\ 0 & 0 & 0\\ 1 & 1 & 1 \end{array}\right],$$

(A4) $$G_{y}=\left[\begin{array}{ccc} -1 & 0 & 1\\ -1 & 0 & 1\\ -1 & 0 & 1 \end{array}\right],$$

The kernels were applied separately to the input image. This allowed producing separate measurements of the gradient component in each orientation. In this case, the gradient magnitude of each orientation was calculated using the Equation (A5):

(A5) $$\left|\nabla \left(x,y\right)\right|=\sqrt{\nabla_{x}^{2}+\nabla_{y}^{2}},$$

After the gradient magnitude image had been computed, pixels with the large gradient magnitude values were considered as edge pixels.

### Appendix A.2. Determining the Facial Symmetry Axis

The convex hull of the edge image is a set of pixels included in the smallest convex polygon that surround all edge pixels and was used to describe the external boundary of the field, where temperature changes were analyzed.

The convex hull of a finite set of points in a plane was computed using the Quick Hull method [16]. First, points with minimum and maximum *x* coordinates were found. These two points were used to form the line dividing the set into two subsets of points, to be processed recursively. In a next step, on one side of the line, the point with the maximum distance from the line was determined. The two points found in the first step along with this new one forms a triangle. Points lying inside of this triangle are not a part of the convex hull and were ignored. This recursive processing was repeated. All points right of the triangle were used as a new subset and everything to the left of it was used as another subset. If the subset only contains the start and end points of the dividing line, then this line must be a segment of the searched hull polygon and the recursion can come to an end. The same operation of forming lines and triangles was repeated until no more points were left: the recursion had come to the end, and the selected points formed the convex hull (see Figure 3).

In the case where the temperature of the chin region is close to the neck temperature, then the final contour of the lower part of the face cannot accurately represent the actual contour. In such a case, the interactive change of a few point coordinates is necessary.

Because the points of the contour do not lie exactly on the ellipse, then we would have m equations of the following general form [31]:

(A6) $$ax_{i}^{2}+bx_{i}y_{i}+cy_{i}^{2}+dx_{i}+ey_{i}+f=r_{i},$$

where *a*, *b*, *c*, *d*, *e*, and *f* are constants of the ellipse polynomial equation, *r_i_* is residual. The system of *m* equations can be written in matrix form as:

(A7) $$\left(\begin{array}{cccccc} x_{1}^{2} & x_{1}y_{1} & y_{1}^{2} & x_{1} & y_{1} & 1\\ \vdots & \vdots & \vdots & \vdots & \vdots & \vdots \\ x_{i}^{2} & x_{i}y_{i} & y_{i}^{2} & x_{i} & y_{i} & 1\\ \vdots & \vdots & \vdots & \vdots & \vdots & \vdots \\ x_{m}^{2} & x_{m}y_{m} & y_{m}^{2} & x_{m} & y_{m} & 1 \end{array}\right)\left(\begin{array}{c} a\\ b\\ c\\ d\\ e\\ f \end{array}\right)=\left(\begin{array}{c} r_{1}\\ \vdots \\ r_{2}\\ \vdots \\ r_{m} \end{array}\right),$$

The problem then is to estimate the model parameters *a*, *b*, *c*, *d*, *e*, and *f* such that the least-squares criterion of residuals *r* is minimized. The criterion is the sum, *S*, of squared residuals, which was calculated as:

(A8) $$S=\sum_{i=1}^{m}r_{i}^{2}\to min,$$

The larger diameter or major axis matches with the symmetry axis of the human face and divides the face TI into left and right sides, as is shown in Figure 3. The center coordinates (*x_c_*,*y_c_*) and clockwise angle *Θ* between the *x*-axis, and the major-axis of an ellipse was calculated [32]:

(A9) $$x_{c}=\frac{cd-bf}{b^{2}-ac},$$

(A10) $$y_{c}=\frac{af-bd}{b^{2}-ac},$$

(A11) $$\theta =\{\begin{array}{cc} 0 & for\;b=0,a<c\\ \frac{1}{2}\pi & for\;b=0,a>c\\ \frac{1}{2}c\tan^{-1}(\frac{a-c}{2b}) & for\;b\neq 0,a<c\\ \frac{\pi }{2}+\frac{1}{2}c\tan^{-1}(\frac{a-c}{2b}) & for\;b\neq 0,a>c \end{array}$$

### Appendix A.3. Detection of Orientation and Position of Mouth Cavity’s Symmetry Axis

The edges of the mouth cavity were also extracted using the convex hull method. It is difficult to fit an ellipse to the mouth cavity contour. So, the *x* position of the symmetry axis was estimated as the centroid of a finite set of contour points using the following equation:

(A12) $$x_{c}=\frac{x_{1}+x_{2}+\cdots +x_{k}}{k},$$

where *x*_1_, *x*_2_, *…x_k_* are *x* coordinates of contour points; *k*—number of contour points. The axis of symmetry is the line that is perpendicular to the *x*-axis that passes through the point *x_c_*, and was used for splitting the mouth cavity into two independent TI regions (see Figure 4).

### Appendix A.4. Calculation of Temperature Differences and of Number of Pixels

The difference between average temperatures of right and left sides of the face or mouth cavity can be expressed as:

(A13) $$\Delta t=\left|\frac{1}{N_{L}}\sum_{i=1}^{N_{L}}t_{Li}-\frac{1}{N_{R}}\sum_{i=1}^{N_{R}}t_{Ri}\;\right|,$$

where $t_{Li}$ and $t_{Ri}$ is the temperature of *i* pixel in left and right side area, respectively; $N_{L}$ and $N_{R}$—the number of pixels in left and right side area, respectively.

The number of pixels in the TI of the right (left) side of the face or mouth cavity having ΔT degree higher temperature than the maximal temperature of opposite side was calculated using histograms of both sides:

(A14) $$n=\{\begin{array}{c} \sum_{i=i_{T_{Lmax}}}^{N_{HL}}n_{Li},\;\;for\;\;T_{Lmax}>T_{Rmax}+\Delta T\;\\ \sum_{i=i_{T_{Rmax}}}^{N_{HR}}n_{Ri},\;\;for\;\;T_{Rmax}>T_{Lmax}+\Delta T\;\\ 0,\;\;\;\;\;\;\;\;\;\;\;\;\;\;\;\;\;\;\;\;\;\;\;\;\;\;for\;\left|T_{Rmax}-T_{Lmax}\right|<\Delta T \end{array}\;,$$

where $\;T_{Rmax},\;T_{Lmax}$—maximal temperature of right and left side, respectively, $i_{T_{Lmax}}$—index of bin having $T_{Lmax}$ temperature, $N_{H}$—number of bins in histogram, and $n_{i}$—number of pixels in i bin. The number of bins $N_{H}\;$ can be calculated from a suggested bin width ∂T as :

(A15) $$N_{H}=\frac{T_{max}-T_{min}}{\partial T},$$

The brackets indicate the ceiling function. In this way, a feature vector has been constructed for each patient.
